# Modeling Time Effects in Anti-Vascular Endothelial Growth Factor Treatment for Diabetic Macular Edema – A Mixed Model Analysis of Real-World Data

**DOI:** 10.7759/cureus.10043

**Published:** 2020-08-26

**Authors:** Ioanna Mylona, Ioannis Tsinopoulos

**Affiliations:** 1 2nd Department of Ophthalmology, Aristotle University of Thessaloniki, Thessaloniki, GRC

**Keywords:** diabetic retinopathy, diabetic macular oedema, anti-vegf treatment, aflibercept, ranibizumab

## Abstract

Objective

The primary objective of this study is to present real-world data on the efficiency of diabetic macular edema (DMO) treatment with anti-vascular endothelial growth factor (anti-VEGF) agents.

Methods

The study included 86 unique eyes from 86 patients who received ≥3 anti-VEGF injections; the mean was 6.49 injections per patient (range 3-10, SD=2.05). The mean age was 66.98 years (SD=9.52) and the mean time in treatment was 12.17 months (SD = 6.5). Data were examined with mixed-effects modeling to account for different time trajectories for treatment in different patients.

Results

DMO treatment had a larger effect size on central retinal subfield thickness than visual acuity. A longer duration of treatment, a shorter time interval between injections, and better baseline values contributed to better treatment outcomes. Patients who start their treatment earlier in the course of the disease will fare better, as will patients who stay the course and keep a sufficient frequency of treatment injections. Patient gender, age, and type of anti-VEGF agent did not have a meaningful effect on the outcomes.

Conclusions

The treatment of DMO with VEGF inhibitors was efficacious and well-tolerated in this clinical practice setting. Maximizing patient involvement, addressing missed appointments due to unforeseen circumstances, and adopting a proactive approach will help ensure positive treatment outcomes for the majority of patients.

## Introduction

Background

Diabetic retinopathy (DR) is the most common microvascular complication of type 1 and type 2 diabetes and is more prevalent in patients who have had diabetes for a longer duration [1]. A meta-analysis for leading causes of vision impairment in higher-income countries and countries in Europe has concluded that DR was the fourth most common cause after uncorrected refractive error, cataract, and glaucoma [2]. DR is caused by chronic hyperglycemia, which results in damage to, and dysfunction of, capillary endothelial cells located in the retina, as well as the other metabolic abnormalities common in diabetes such as diabetic dyslipidemia, hypertension, and vascular inflammation [1,3]. DR is classified into several stages based on the level of disease severity, including pre-retinopathy, mild non-proliferative DR (NPDR), severe NPDR, proliferative DR, and diabetic macular edema (DMO); the latter three stages being classified as vision-threatening diabetic retinopathy (VTDR). DMO is an advanced, vision-limiting manifestation of DR, in which swelling of the central retina causes loss of central vision. It affects one in 15 people with diabetes, resulting in more than 20 million cases worldwide [4]. A meta-analysis of 35 studies published between 1980 and 2008, which included a total of 22,896 individuals with DM, concluded that the overall prevalence was 34.6% (95% CI 34.5-34.8) for any DR, 6.96% (6.87-7.04) for proliferative DR, 6.81% (6.74-6.89) for diabetic macular edema, and 10.2% (10.1-10.3) for VTDR [5]. Risk factors for diabetic retinopathy include an increase in glycated hemoglobin A1c (meaning poor glycemic control), increased systolic blood pressure, hyperlipidemia, and increased body mass index post-puberty and pregnancy [6]. Unfortunately, even patients in the later stages of the disease may be asymptomatic, leading to severe vision loss, thus necessitating a high degree of clinical vigilance and frequent retinal examinations for DR patients [1].

Treatment of DMO

Today's standard of care includes pharmacotherapy with anti-vascular endothelial growth factor (anti-VEGF) agents that replaced focal or grid laser photocoagulation to a large extent. VEGF is a potent, diffusible, endothelial-specific mitogen that mediates many important physiologic processes, including the development and maintenance of vasculature, the regulation of blood coagulation and vascular tone through the production of nitric oxide and prostacyclin I2, the regulation of the podocytes necessary for glomerular filtration by the kidneys, and the maintenance of the integrity of epithelial cell layers during normal wound repair [3]. A recent Cochrane database report concluded that anti-VEGF agents were all more effective than laser for improving vision by 3 or more lines after one year, with approximately one in 10 people improving vision with laser, and about three in 10 people improving with anti‐VEGF treatment [7].

Although anti-VEGF therapy is more efficacious, there are drawbacks; patients are required to have a greater number of visits and receive a greater number of treatments than is typically required for management with pan-retinal laser, which may not be optimal for some patients [1]. Patients are required to undergo the discomfort and inconvenience of regular injections in order to preserve and improve their vision, and although the trade sounds fair, in the real world, this is not always the case. Also, problems with physical health may lead to missed or rescheduled appointments. A recent study looked at the habits of 136 individuals with DMO and 109 with age-related macular degeneration (AMD). The researchers showed that 46% of DMO patients and 22% of AMD patients had at least one absence of more than 100 days while only 35% of DME patients and 50% of AMD patients were always on schedule. In 60% of DMO cases that went more than 100 days without follow-up, the patient’s visual acuity was worse than that before they ceased treatment [8]. The most frequently stated reason for missed appointments was other health issues. In view of those real-world data, it comes as no surprise that a multi-center evaluation of anti-VEGF treatment for DMO concluded that anti-VEGF injections were administered less frequently and were less effective than those in the agent registration trials [9]. However, missed appointments may be addressed by an appropriate treatment plan. Results from a related study that only included patients with AMD suggests that while missed hospital appointments may be a relatively common occurrence, good outcomes of treatment can be achieved over two years despite missed hospital visits if patients are reviewed on average six times in the first year after an initial loading phase of three injections and nine times in the second year of treatment [10].

Aim of the study

The aim of this study is to analyze real-world data from anti-VEGF treatment for DMO, in order to assess treatment efficacy in a real-world environment while controlling for possible confounding factors.

## Materials and methods

Study setting and goals

This is an observational study with all patients already undergoing treatment for DMO in the 2nd Department of Ophthalmology, Medical School, Aristotle University of Thessaloniki and had a scheduled visit from January to May 2019. The study included 86 unique eyes from 86 patients who had already received ≥3 anti-VEGF injections; the mean was 6.49 injections per patient (range 3-10, SD=2.05). The mean age was 66.98 years (SD=9.52), and the mean time in treatment was 12.17 months (SD = 6.5). Fifty-two patients (60.5%) received aflibercept treatment and 34 patients (39.5%) ranibizumab treatment. Visual acuity (VA) was assessed on the Early Treatment Diabetic Retinopathy Study (EDTRS) scale while central subfield thickness (CST) was measured by way of optical coherence tomography (OCT) in μm. Table 1 presents sample demographics in total and by gender, number of injections received, involved eyes, duration of treatment, initial/final visual acuity measured with the EDTRS system (VA), and initial/final central retinal subfield thickness (CST).

**Table 1:** Participants’ characteristics at baseline and end VA: visual acuity; CST: central subfield thickness

Participants	Overall (86)	Male (59)	Female (27)	p value
Age in years (SD)	66,98 (9.52)	66.34 (10.18)	68.4 (7.89)	0.353
Number of injections (SD)	6.49 (2.05)	6.66 (2.07)	6.11 (1.98)	0.251
Time in treatment	12.17 (6.5)	11.86 (6.29)	12.84 (7)	0.521
Eye	OD 46 (53.5%) OS 40 (46.5%)	OD 32 (54.2%) OS 27 (45.8%)	OD 14 (51.9%) OS 13 (48.1%)	0.837
VA (start)	0.584 (0.264)	0.569 (0.254)	0.617 (0.285)	0.437
CST (start)	407.232 (120.203)	414.017 (133.086)	392.407 (85.98)	0.442
VA (end)	0.548 (0.271)	0.527 (0.271)	0.55 (0.275)	0.72
CST (end)	332.244 (101.754)	327.305 (108.781)	343.037 (85.296)	0.509

The study has been approved by the Ethical Committee of the Aristotle University of Thessaloniki in accordance with the recommendations of the Declaration of Helsinki for studies involving human subjects.

Statistical analysis

Comparisons on demographic variables were carried out with the Pearson's chi-square test and the student's t-test. Simple comparisons were carried out between pre- and post-intervention measurements with the Pearson's t-test for paired samples. The analysis of real-world data has unique requirements since the number of instances for each individual differs. One of the preferred methodologies on this occasion is mixed-effects modeling. In mixed-effects modeling, the joint consideration of fixed and random effects estimates both a subject-specific baseline for the outcome and a subject-specific trend (over time) for the explanatory variables, which allows the extent of inter-individual variations to be measured. Random effects can be assumed on any covariate or any cluster of subjects to capture correlated characteristics in the data; fixed-effects estimates are interpreted as the conditional effects in the presence of the covariates with random effects [11].

The procedure calls for the incremental assessment of nested models, where parameters are added and output compared for accuracy and parsimony by way of fit indexes (such as the log-likelihood or Akaike's Information Criterion (AIC), where lower values are better). The first model only included the intercept and provided a baseline. The second model included the effect of the injections and the elapsed time in months while the third model also included an interaction effect between the number of injections and the elapsed time. The models included a random effect for the duration of treatment with an unstructured covariance structure and a diagonal covariance effect for the repeated measures effect of the number of injections. Alternative models assessed the possible effect of age both as a fixed and a random effect, and gender as a fixed effect but none were statistically significant.

All group comparisons were computed with the IBM Statistical Package for the Social Sciences (SPSS) (version 25; IBM Corp., Armonk, NY) and the mixed model's assessment with the PROC MIXED procedure of the same statistical package.

## Results

There were no notable differences between the genders on the sample demographics. Comparisons between the pre and post-intervention measurements with the Pearson's t-test for paired samples revealed that there were statistically significant differences before treatment initiation and after the latest injection, for VA p = 0.012 and for CST p < 0.001 (Table 2).

**Table 2:** Comparisons on visual acuity (VA) and central retinal subfield thickness (CST) on treatment initiation and after the last received injection

	Paired Differences					t	df	p	Effect size (d)
	Mean	Std. Deviation	Std. Error Mean	95% Confidence Interval of the Difference					
				Lower	Upper				
VA before – VA after	0,049	0,177	0,019	0,011	0,087	2,582	85	0,012	0.278
CST before – CST after	74,988	97,902	10,557	53,998	95,978	7,103	85	<0.001	0.766

Effect sizes were 0.278 for VA, a medium effect size, and 0.766 for CST, a very large effect size denoting considerable clinical significance.

**Figure 1:**
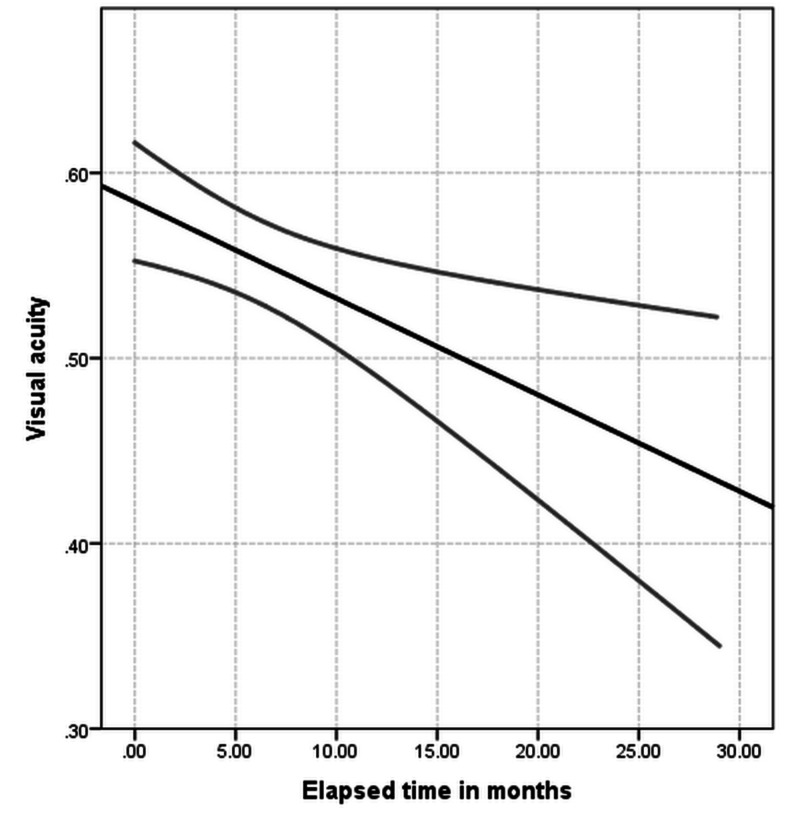
Mean values for visual acuity over time including 95% confidence intervals

**Figure 2:**
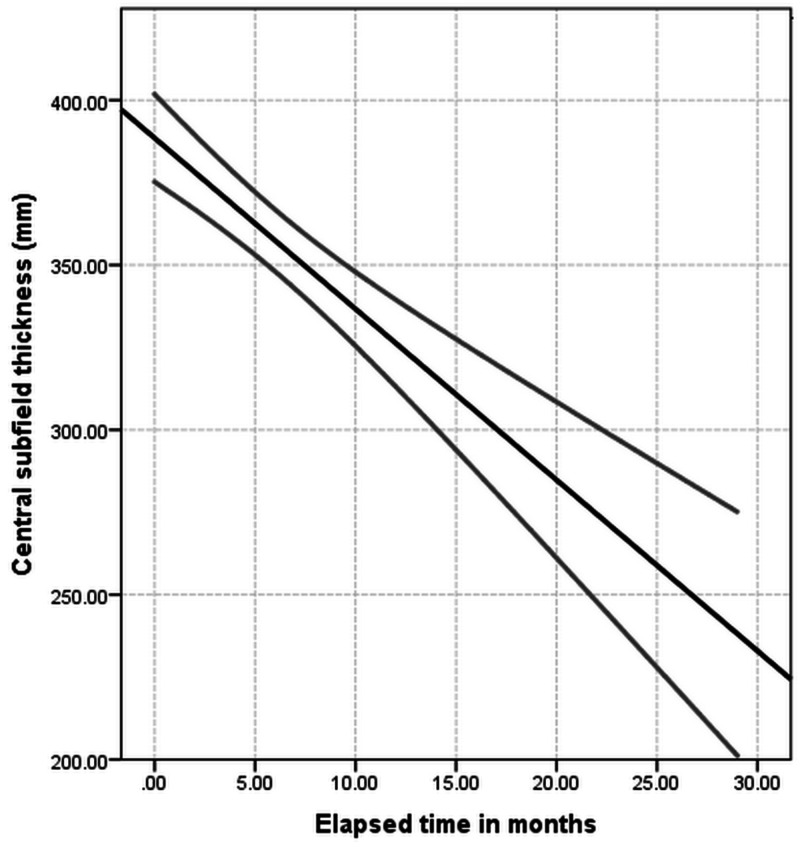
Mean values for central subfield thickness over time including 95% confidence intervals

Figures 1-2 present the course of improvement over time, including confidence intervals.

Also, of note, there were no reports of adverse events related to the injection procedure, with treatment being well-tolerated in the clinical practice setting.

In the next step, the aim was to construct a model that best accounts for those evident improvements over the treatment course while taking into account a contribution of possible confounders and contributors, including random effects. To this extent, a series of linear mixed models with increasing degrees of adjustment (nested models that differed between them by a parameter) were tested against the data and compared for parsimony and accuracy with the fit indices.

Each model was tested against data for VA and CST separately (dependent variables). The first model is intercept-only and serves as a baseline. The second model included linear trajectories in VA and CST with the duration of treatment for each participant at the first level and variations in VA and CST trajectories across participants adjusted for the fixed effects of the number of injections received and the total duration of treatment. The third model further included the fixed effect of the number of injections received over the duration of treatment (frequency of injections). A fourth model included other potential mediators, including the effect of the age of the subject at baseline, the time-varying effect of age, the effect of the type of injection (aflibercept vs ranibizumab). None of the additional factors tried in model 4 (age at baseline, the time-varying effect of age, type of injection) had any statistically significant effect and the resulting models had worse fit indexes than model 3 for VA and model 2 for CST.

While assessing VA, the third model returned better fit indexes while the second model was more suitable in CST assessment. In VA measurement, the fixed effect for the number of injections was statistically significant (p = 0.007) as was the combined effect of the number of injections by the duration of treatment (p = 0.037) but not the duration of time on its own (p= 0.699). In the case of CST measurement, the second model had a statistically significant effect for the number of injections (p <0.001) and a significant effect for the duration of treatment (p =.047). While examining the covariance structure of each model's random effects, there were statistically significant effects in each case, showing that total treatment time had a statistically significant effect (p<.001 for VA and CST) as did the time interval between individual injections (negative effect, p=0.028 for VA, and p <0.001 for CST). Initial VA and CST levels were positively linked to the improvement during the treatment (p<0.001 in both cases). The effect of the injections was, in both cases, statistically significant; for VA univariate F (8, 19.505) = 3.531, p = 0.011 and for CST univariate F (9, 52.255) = 8.882, p < 0.001. Tables 3-4 present the results for the tests on the fixed effects for VA and CST, respectively.

**Table 3:** Tests of fixed effects for model 3 for visual acuity

Source	Numerator df	Denominator df	F	p
Intercept	1	217.165	158.860	<0.001
Injections	9	22.894	3.539	0.007
Duration of treatment	1	225.662	.150	0.699
Injections * Duration of treatment	8	30.817	2.418	0.037

**Table 4:** Tests of fixed effects for model 2 for central retinal subfield thickness

Source	Numerator df	Denominator df	F	p
Intercept	1	292.889	243.223	<0.001
Injections	9	51.570	8.882	<0.001
Duration of treatment	1	189.264	3.813	0.047

## Discussion

The primary objective of this study was to present real-world data on the efficiency of the DMO treatment. DMO treatment had significant gains when comparing pre- and post-treatment visual acuity and central retinal subfield thickness. We also assessed the longitudinal change with treatment so as to discern the impact of demographic variables and treatment variables that cannot be excluded when examining data that do not adhere to the strict inclusion criteria of a typical closed efficacy study.

The study confirmed that even in this uncontrolled clinical setting, pre-treatment scores showed a statistically significant improvement after DMO treatment initiation. Regarding confounding variables, the final models showed that a few tested variables had a meaningful effect; those were the number of injections received and the duration of treatment for CST, while for VA, it was the number of injections and the combined effect of that number with treatment duration (higher frequency of injections). When examining random individual effects for both outcomes, it was determined that a lengthier duration of treatment, a shorter time interval between injections, and better baseline values, all contributed to better treatment outcomes. These results demonstrate the benefit of a well-thought-out treatment plan and an early initiation of treatment. There were no significant effects for the subjects’ age, gender, or the time-varying effect of age (meaning further macular degeneration during treatment). Also, there was no notable difference between the two different treatment modalities (aflibercept vs ranibizumab). We should note that their choice for any particular patient was made exclusively with clinical considerations, as is the case with real-world data.

Although data indicated that there were clear benefits on the whole when comparing pre-treatment status to current status, there were also a number of poor respondents, including both patients who were under treatment for a significant amount of time and patients in early treatment. Poor initial treatment response may lead to poorer compliance and further reduce the odds of eventual response to treatment while patients who miss appointments may appear to make little progress despite being on treatment for a long period of time. This reality shows the necessity for a personalized treatment plan. A recent meeting of a global group of ophthalmic experts (Vision Academy Steering Committee) identified four key principles for the ideal treatment regimen for anti-VEGF management of retinal diseases, including maximizing and maintaining visual acuity (VA) benefits for all patients, deciding when to treat next, rather than whether to treat now, titrating the treatment intervals to match patients’ needs and treating at each monitoring visit [12]. It is also proposed that the adoption of a proactive and more personalized approach in the clinic such as a treat-and-extend regimen will lead to benefits for both the patient and the physician, through a reduction in the associated treatment burden and better utilization of clinic resources. Results from this study confirm that a tight schedule of anti-VEGF injections is beneficial as is the maximizing of treatment duration, goals that will require patient commitment and full co-operation, as well as the ability to flexibly reschedule appointments as close to the original appointment as possible, in the event of an unrelated health event that requires attention.

Limitations

A significant limitation of this study is the lack of recorded reasons for any missed appointments and rescheduling; more care should be taken to address this significant issue and promote a better understanding of potential obstacles for patients when scheduling appointments, outside random events. We should also note that the positive outcomes in this study cannot be generalized on the entire patient population who are potential candidates for anti-VEGF treatment since those patients who were included in our study had received at least three anti-VEGF injections. Potential candidates for anti-VEGF treatment who did not respond to the initial two injections with reduced edema or improved vision or who have had an adverse reaction to the injection procedure would have discontinued treatment, as per the standard protocol.

## Conclusions

Treatment of DMO with VEGF inhibitors was efficacious and well-tolerated in this clinical practice setting. Patients who start their treatment earlier in the course of the disease will fare better, as will patients who stay the course and keep a sufficient frequency of treatment injections. Maximizing patient involvement, addressing missed appointments due to unforeseen circumstances, and adopting a proactive approach will help ensure positive treatment outcomes for the majority of patients.
